# Coexistence of mucinous nevus and multiple collagenoma with unilateral, dermatomal, and multi segmental distribution

**DOI:** 10.1002/ccr3.7429

**Published:** 2023-06-13

**Authors:** Gita Faghihi, Reza Moeine, Fatemeh Mohaghegh, Parvin Rajabi, Reza Shahriarirad

**Affiliations:** ^1^ Department of Dermatology, School of Medicine Isfahan University of Medical Sciences Isfahan Iran; ^2^ Thoracic and Vascular Surgery Research Center Shiraz University of Medical Sciences Shiraz Iran

**Keywords:** coexistence, collagenoma, histopathologic, mucinous nevus

## Abstract

Connective tissue nevus is a hamartoma composed of excess amounts of one or several components of the dermis, such as collagen, elastin, and proteoglycans. This report introduces a 14‐year‐old girl with grouped flesh color papules and skin color nodules distributed unilaterally with a dermatomal pattern. These lesions involved more than one segment. Histopathology is the gold standard for diagnosing collagenoma and mucinous nevus. We reported the first case of mucinous nevus with multiple collagenoma that shows the specific clinical features.

## Graphical abstract

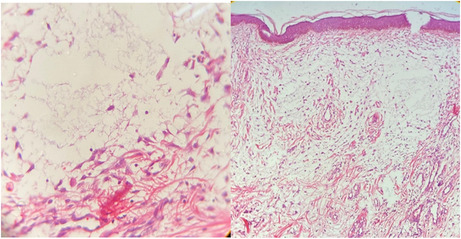

## 1 BACKGROUND

Connective tissue nevi are hamartomas of the dermis, with the three main components of collagen (collagenoma), elastin (elastoma), and proteoglycans (mucinous nevus). Each subtype can present as solitary or multiple lesions.^1^ Collagenomas are connective tissue nevi that represent hamartomatous proliferation of collagen, which is defined based on their pattern of distribution (localized or generalized) and mode of inheritance (acquired or inherited).^2^ Mucinous nevus is a rare entity first described by Redondo Bello'n et al. in 1993 that is divided into two histopathologic types as connective tissue nevus of the proteoglycan and combined epidermal‐connective tissue nevus of the proteoglycan.^3^

## 2 CASE PRESENTATION

The patient was a 14‐year‐old girl with a chief complaint of rashes and itching on her body and extremities. She stated that her problem started a year and a half ago. She denies any significant past medical or medical history or any similar history of skin disorders in her family.

**FIGURE 1:**
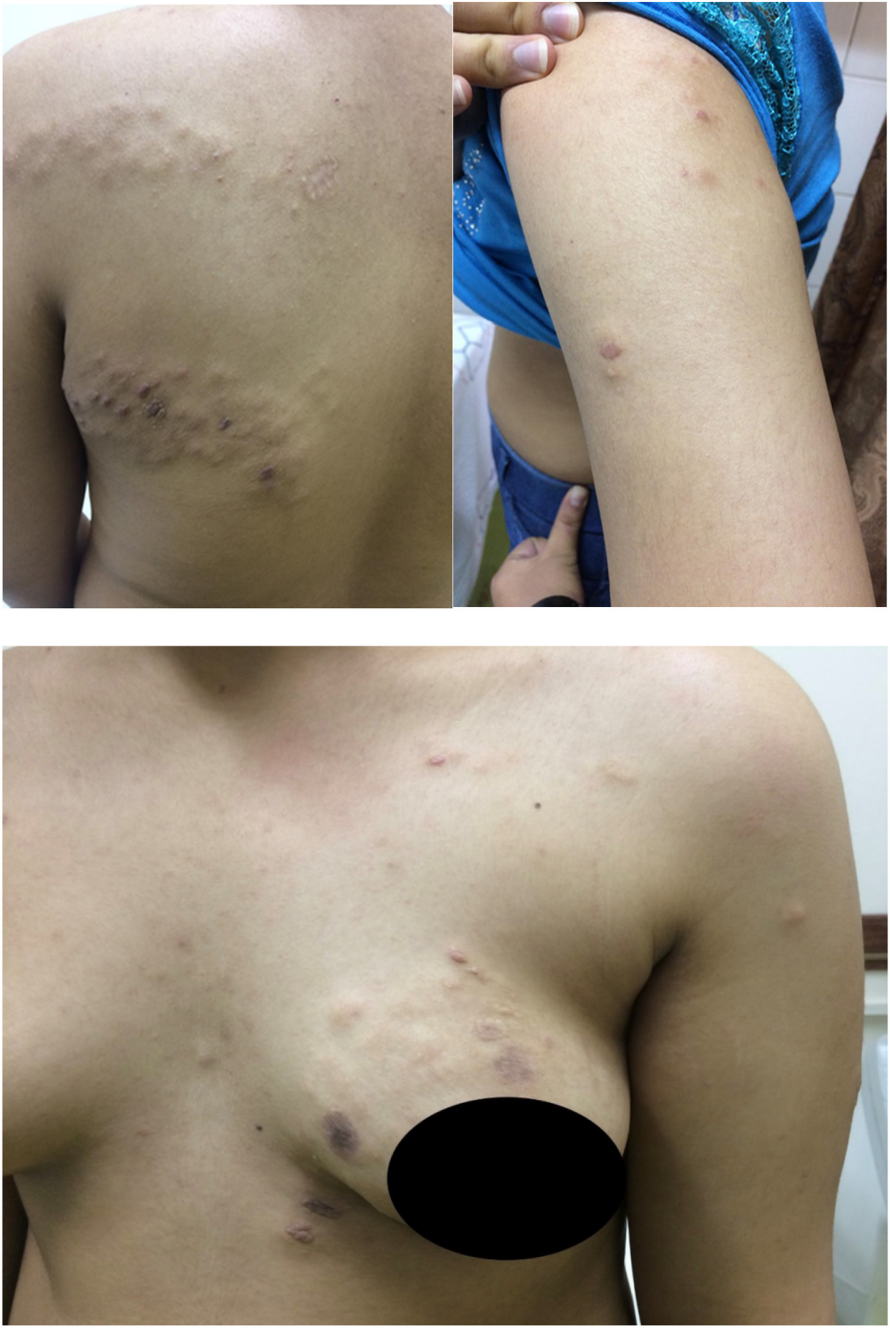
A 14‐year‐old girl with grouped flesh color papules and skin color nodules distribute unilateral and multi‐segment with dermatomal patterns on the upper back, breast, chest, and upper limbs.

On physical examination, skin involvement was in the form of grouped flesh color papules and skin color nodules distributed unilateral and multi‐segment with dermatomal patterns on the upper back, breast, upper chest and upper limbs, which were also pruritic. (Figure 1) The lesions were initially in her upper back and chest, which eventually spread to the proximal upper limb and fewer lesions in the distal upper limb within a year and a half, which was then halted. There was no evidence of angiofibroma or hyperpigmented lesions or any other dermatome involvements or similar lesions. The patient also did not have any signs or history of rheumatological disorders, joint involvement, or any history of seizures.

**FIGURE 2:**
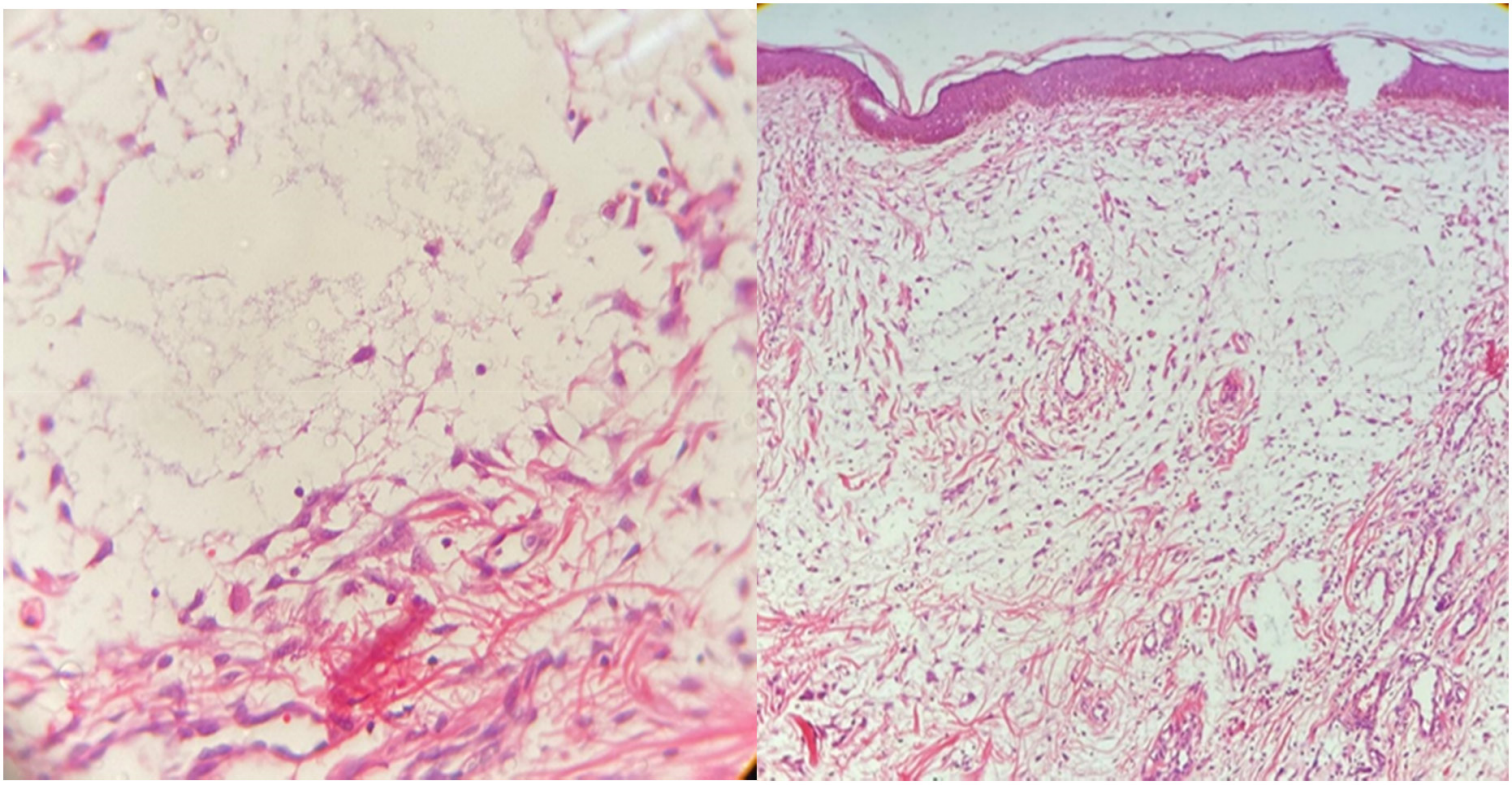
Deposition of amorphous materials in the high‐power field.

**FIGURE 3:**
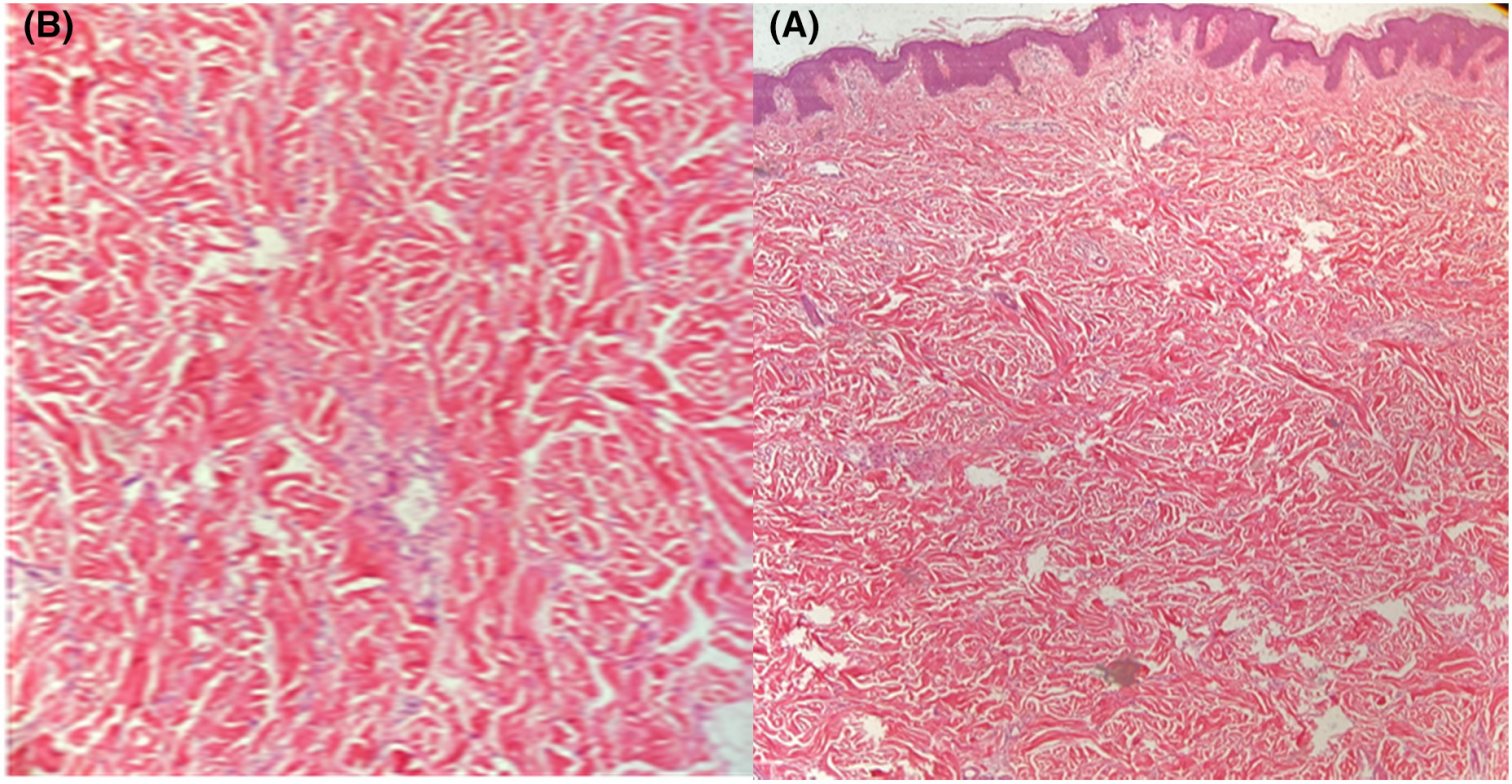
(A) Separation of collagen bundles by mucin. (H & E *40); (B) Fibroblasts with stellate forms (H & E *100).

**FIGURE 4:**
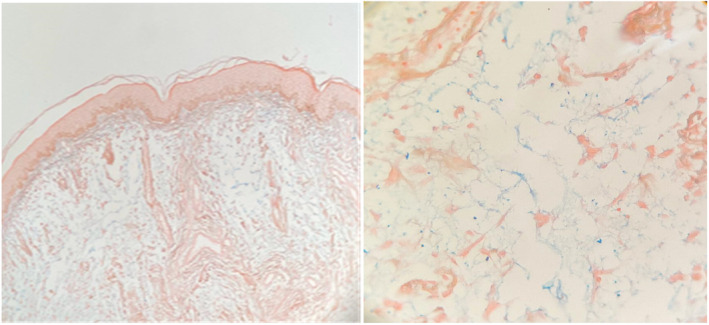
Increased mucin throughout the papillary dermis. Mucin deposition is confirmed with a mucin stain. (Alcian blue).

Two biopsies from both papular and nodular lesions were performed; the differential diagnoses were leiomyoma, adnexal tumor, and collagenoma. The specimens from the papular lesion showed a normal epidermis with an “empty appearance” due to the deposition of amorphous materials, which separated collagen fibers in the papillary dermis. Fibroblasts were increased in number, and an inflammatory infiltrate composed of lymphocytes and mast cells was seen in the mid‐dermis beneath the material deposition (Figure 2). Alcian blue stain confirmed mucin deposition in the papillary dermis, and biopsy specimens of nodular lesions showed spread presence of collagen bundles in the dermis (Figure 3). There was increased mucin throughout the papillary dermis, which was confirmed with a mucin stain. (Alcian blue) (Figure 4).

Results of laboratory studies, including complete blood cell count, liver and renal function tests, radiologic examination of the chest and lower extremities, and echocardiography, were normal. Considering the clinical and pathologic findings, a diagnosis of mucinous nevi with multiple collagenoma in dermatomal and unilateral distribution was rendered.

## 3 DISCUSSION

Cutaneous mucinoses are a heterogeneous group of disorders associated with an abnormal amount of mucin in the skin.^4^, ^5^ The term “mucinous nevus” was proposed because of its nevoid appearance and characteristic pattern of mucin deposits in the papillary dermis.^6^ Connective tissue origin will further divide mucinous nevi into two histopathologic types of connective tissue nevus of the proteoglycan and combined epidermal‐connective tissue nevus of the proteoglycan.^1^ In connective tissue nevus of the proteoglycan, the epidermis is normal, while in epidermal‐connective tissue nevus of the proteoglycan, the epidermis shows hyperkeratosis and acanthosis with elongation of the rete ridges consistent with an epidermal nevus. As epidermal changes were not found in our case, we diagnosed connective tissue nevi of the proteoglycan type.^3^

The mucin is thought to be composed of hyaluronic acid staining positively with alcian blue at a pH of 2. In the present case, mucin deposits were present in the papillary dermis.^7^ Collagenomas are connective tissue nevi representing hamartomatous and predominantly composed of collagen. Inherited collagenomas are autosomal dominantly inherited and include familial cutaneous collagenomas, Shagreen patch of tuberous sclerosis and Buschke–Ollendorff syndrome. Acquired collagenomas include eruptive collagenoma and isolated collagenoma.^8^ The typical clinical presentation of mucinous nevus is grouped brownish papules and confluent plaques^9^ while the clinical presentation of collagenomas includes skin‐colored papules, nodules of different sizes single or multiple arranged in a linear or segmental pattern.^4^, ^10^ In our case, the lesions were flesh color papules and skin color nodules, with predominant nodular lesions.

There was no evidence of angiofibroma, hyperpigmented lesions, or any other dermatome involvements or similar lesions as mucinous lesions sometimes occur in the context of other skin diseases, such as vascular melange. The patient also did not have any signs or history of rheumatological disorders, joint involvement, or any history of seizure as collagenoma is occasionally related to hereditary diseases such as tuberous sclerosis.

To our knowledge, no cases of mucinous nevus with collagenoma have been reported in Iran. The most common site of lesions is the upper trunk, arms, back and thighs, with the most common configuration of linear or zosteriform or grouped distribution.^4^, ^5^ In our case, the lesions were also distributed on the back, but there were in the proximal and distal upper limbs, and the pattern of lesions was dermatomal, and two dermatomes were involved. This pattern of skin lesions seems to be due to the cell's migration to specific areas of the skin during embryonic development and after the post‐zygotic mutation. This mutation makes a colony of cells susceptible to genetic or acquired dermatosis.^11^ The differential diagnoses are leiomyoma and adnexal tumor and the clinical features of a mucinous nevus with collagenoma, which are indistinguishable from leiomyoma and adnexal tumor but can be distinguished by histopathological features.^12^, ^13^

There is no need for treatment due to the presence of mucus nevus lesions, except for cosmetic purposes. Surgical excision, dermabrasion, and carbon dioxide have been used for treatment.^3^ Satisfactory treatment of solitary collagenoma with intradermal steroid injection has been reported, and surgical excision may also be used.^14^, ^15^, ^16^, ^17^

## 4 CONCLUSION

We reported the first case of mucinous nevus with multiple collagenoma that showed specific clinical features. Histopathology is the gold standard for the diagnosis of collagenoma and mucinous nevus.

## AUTHOR CONTRIBUTIONS

**Gita Faghihi:** Conceptualization; supervision. **Reza Moeine:** Conceptualization; data curation. **Fatemeh Mohaghegh:** Supervision; validation. **Parvin Rajabi:** Data curation; writing – original draft. **Reza Shahriarirad:** Writing – original draft; writing – review and editing.

## FUNDING INFORMATION

Not applicable.

## CONFLICT OF INTEREST STATEMENT

The authors have no conflict of interest to declare.

## ETHICAL APPROVAL

Written informed consent was obtained from the patient's legal guardian or next of kin for publication of this case report and any accompanying images. A copy of the written consent is available for review by the Editor‐in‐Chief of this journal.

## CONSENT

Written informed consent was obtained from the patient's legal guardian for publication of this case report and any accompanying images. A copy of the written consent is available for review by the Editor‐in‐Chief of this journal.

## Data Availability

All supporting data is available and have been reported in the manuscript. Please contact the corresponding author in case of requiring any further information.
